# Isoalantolactone Enhances the Radiosensitivity of UMSCC-10A Cells via Specific Inhibition of Erk1/2 Phosphorylation

**DOI:** 10.1371/journal.pone.0145790

**Published:** 2015-12-30

**Authors:** Yonggang Fan, Zhiyong Weng, Hongguo Gao, Jiehua Hu, Hongyan Wang, Lihua Li, Hua Liu

**Affiliations:** 1 Department of Cell Biology, Liaoning Medical University, Jinzhou, Liaoning, China; 2 Department of clinical laboratory, Daqing oilfield general hospital, Daqing, Heilongjiang, China; 3 Information Center, Logistics college, Naval University of Engineering, Tanggu, Tianjin, China; National Cheng Kung University, TAIWAN

## Abstract

**Background:**

Although radiotherapy is one of the mainstream approaches for the treatment of head and neck squamous cell carcinoma (HNSCC), this cancer is always associated with resistance to radiation. In this study, the mechanism of action of isoalantolactone as well as its radiosensitizing effect was investigated in UMSCC-10A cells.

**Methods:**

The radiosensitization of UMSCC-10A cells treated with isoalantolactone was analyzed by colony formation assay. The radiosensitization effects of isoalantolactone on cell proliferation, cell cycle and apoptosis regulation were examined by BrdU incorporation assay, DNA content assay and flow cytometry, respectively. Western blotting was performed to determine the effects of isoalantolactone combined with radiation on the protein expression of Mek, extracellular signal-regulated kinase (Erk1/2) as well as phosphorylated Mek and Erk1/2. Erk1/2 knockdown by siRNA was used to confirm that isoalantolactone specifically inhibited the activation of Erk1/2 signaling pathway in UMSCC-10A cells.

**Results:**

Isoalantolactone enhanced the radiosensitivity of UMSCC-10A cells; the sensitivity enhanced ratios (SERs) were 1.44 and 1.63, respectively, for 2.5 and 5 μM. Moreover, isoalantolactone enhanced radiation-induced cell proliferation and apoptosis and cell cycle arrested at G2/M phase. Furthermore, no marked changes were observed in the expression of total Erk1/2 and Mek protein after radiation treatment. However, isoalantolactone was significantly reduced radiation-induced the phosphorylation of Erk1/2, whereas it altered the phosphorylation of Mek to a lesser extent. In addition, the radiosensitivity of UMSCC-10A cells with Erk1/2 knockdown was increased. Isoalantolactone cannot further prevent the proliferation of UMSCC-10A cells with Erk1/2 knockdown which other mechanism regulated cell proliferation.

**Conclusion:**

Our results suggested that isoalantolactone enhanced radiation-induced apoptosis, cell cycle arrested and reduced the cell proliferation of UMSCC-10A cells via specifically inhibited the phosphorylation of Erk1/2. Thus a low concentration of isoalantolactone may be used to overcome the resistance of UMSCC-10A cells to radiation and may be a promising radiosensitizer in cancer therapy.

## Introduction

Human head and neck cancer is the sixth most common form of cancer worldwide [1], and of the various types, 90% of cases are head and neck squamous cell carcinomas (HNSCCs) [2]. Due to its significant morbidity and mortality rates, HNSCC is a devastating malignant tumor [3]. At present, surgical abscission, chemotherapy and radiotherapy are the most frequent methods used to treat this disease [4, 5]. Specifically, radiotherapy plays an important role in the treatment of this disease since the symptoms associated with HNSCCs tend to appear very late, and therefore, patients are often diagnosed at an advanced stage. However, radiation alone does not contribute significantly in terms of a cure for HNSCC, and it has the disadvantage of significant side effects [6, 7]. It is worth noting that the overall 5- year survival rate has only been 50% during the last few decades [8]. Therefore, the identification of a substance with the ability to specifically sensitize tumor cells to radiotherapy as well as an understanding of the molecular mechanisms would have far-reaching consequences and would lead to more effective anticancer therapies [9].

The extracellular signal-regulated kinase Erk1/2 pathway is a classical cell signaling pathway, as it links extracellular signals and membrane-based receptors that regulate many cellular functions, such as gene expression, cell growth, differentiation, survival and apoptosis [10, 11]. Abnormal Erk1/2 signaling may lead to increased or uncontrolled cell proliferation, resistance to apoptosis and resistance to chemotherapy, radiotherapy, and targeted therapies in tumors [12, 13]. Moreover, previous studies have shown that low-dose radiation can promote cell growth and proliferation as a way to avoid the stress of radiation; this has been associated with the activation of the Erk1/2 signaling pathway in normal and tumor cells [14, 15]. Recently, activation of the Erk1/2 signaling pathway was found to contribute to the effects of radiation resistance in many tumor cells [6, 16]. According to these findings, blockage of Erk1/2 pathway activity may significantly improve the response of tumor cells to radiotherapy. Thus, this pathway will be a potential target for improved radiosensitivity outcomes of tumor therapy.

Isoalantolactone, a sesquiterpene lactone compound that can be purified from the roots of *Inula helenium L*, has long been used in Chinese traditional medicine. Isoalantolactone possesses many pharmacological and biological activities, such as antifungal, anti-bacterial, anti-helminthic and anti-proliferative properties [17]. Recently, we and others have discovered that isoalantolactone exerts powerful antitumor effects in gynecologic tumors [18], pancreatic cancer [19], human HNSCC [20] and gastric cancer [21]. Mechanistically, isoalantolactone induces cell apoptosis through the production of reactive of oxygen species and the repression of the activation of the PI3K/AKT signaling pathway. However, it is unclear whether isoalantolactone has the ability to enhance the radiation sensitivity of tumor cells of any type. In the present study, the effects and the molecular mechanism of a combination of isoalantolactone and radiation were investigated in HNSCC cell lines.

## Materials and Methods

### Reagents

Isoalantolactone was purchased from the National Institution for the Control of Pharmaceutical and Biological Products in China, and its purity (>99%) was defined by HPLC. Isoalantolactone was dissolved in dimethylsulfoxide (DMSO) to a 20 mM stock solution, which was stored at -20°C and diluted to the desired final concentration in DMEM medium at the time of use. Propidium iodide (PI), dimethylsulfoxide (DMSO), [3-(4,5-dimethylthiazol-2-yl)-2,5-diphenyltetrazolium bromide] (MTT), Dulbecco’s Modified Eagle’s Medium (DMEM), fetal bovine serum (FBS), RNase A, BrdU, penicillin and streptomycin were purchased from Sigma Chemical Co. (USA). The annexin V-FITC apoptosis detection kit was purchased from Beyotime Institute of Biotechnology (China). On-TARGETplus SMARTpool siRNA for Erk1/2 kit was purchased from Dharmacon (USA). The Lipofectamine 2000 kit was purchased from Invitrogen (USA). Primary antibodies were purchased from Cell Signaling Technology (USA), whereas antibodies specific to β-actin and horseradish peroxidase-conjugated secondary antibodies were purchased from Invitrogen (USA).

### Cell Culture

The UMSCC-10A, UMSCC-12, Cal-27 and HepG2 cell lines were purchased from the Shanghai Institute of Biological Science in China. The cells were cultured in DMEM medium supplemented with 10% fetal bovine serum, 100 U/ml penicillin and 100 μg/ml streptomycin and were incubated at 37°Cin a humidified atmosphere of 5% CO_2_. Fisher rat thyroid (FRT) cell line was purchased from American Type Culture Collection (ATCC) in USA. The cells were cultured at 37°C (5% CO_2_) with Coon’s F-12 growth medium containing 10% FBS, 100 U/ml penicillin, 100 μg/ml streptomycin, 4 mM L-glutamine. The medium was changed twice per week. Cell detachment was achieved by the addition of a 0.05% trypsin/0.02% EDTA solution.

### Cell proliferation assay

For analysis of DNA synthesis, cells were seeded into 6-well culture plates. After the cells attached, the cells were treated with radiation (4 Gy) for 4 hours and 5 μM isoalantolactone either individually or in combination. After 72 hours, cells were incubated for 60 minutes with 10 μM BrdU, fixed in 4% paraformaldehyde, and permeabilised with 0.1% Triton X-100. Then cells were incubated with 2 N HCl for 30 minutes at 37°C and with 0.1 M borate buffer (pH 8.5) for 10 minutes at room temperature. After blocking with 2% BSA, they were incubated with an anti-BrdU antibody overnight at 4°C, followed by reaction with FITC-labeled anti-mouse IgG. At least 300 cells per slide were examined in three randomly selected high-power fields, and the percentage of positive staining was calculated.

### Radiation exposure and clonogenic survival assay

Cells were seeded into 6-cm culture dishes. After the cells attached, the cells were incubated with isoalantolactone (2.5 or 5 μM) for 16 hours, and subsequently, the cells were radiated with a single boost of 0, 2, 4, 6 or 8 Gy using a conventional radiation source with a 160-kV X-ray machine (RAD SOURCE, Suwanee, GA). Four hours after radiation, the culture medium was changed. After 14 days, the colonies were stained with coomassie blue, and the number of colonies with at least 50 cells was counted using Image-Pro Plus sofeware. The survival fraction (SF) was calculated as the ratio of the number of colonies to the number of cells per plate in each treatment group divided by the plating efficiency of the control group. In addition, after radiation treatment (0, 7, 15 days), the cells were counted with trypan blue staining in a cell viability analyzer (Beckman Coulter, Epics XL, USA) in order to determine the cell viability. All experiments were repeated at least 3 times.

### Apoptosis and cell cycle analysis

In all, 5×10^5^cells were seeded in 6-well plates. After 24 hours, the cells were treated with radiation (4 Gy) and 5 μM isoalantolactone either individually or in combination. In regards to the combination treatment, isoalantolactone at a dose of 5 μM was added to the cultured cells 16 hours prior to radiation exposure. The cells were then washed with PBS and were subsequently resuspended in 200 μl binding buffer supplemented with 5 μl annexin V (10 μg/ml) for 10 minutes in the dark. Then, the cells were incubated with 10 μl PI (20 μg/mg), and the samples were almost immediately screened for apoptosis using flow cytometry (Beckman Coulter, Epics XL, USA). CellQuest software was used for data collection and analysis [21].

In regards to the cell cycle analysis, the cells were treated as described above, and were then collected and fixed in 70% ethanol for 2 hours. This was followed by incubation with PI (20 μg/ml) and ribonuclease (200 μg/ml) for 30 minutes at 37°C. The DNA content was analyzed by flow cytometry, and all samples should have contained at least 10,000 cells. The data were analyzed using CellQuest software.

### siRNA silencing of Erk1/2

In all, 5 ×10^5^ cells were seeded in 6-well plates. After 24 hours, the cells were transfected with siRNA using Lipofectamine 2000. After 3 days, the transfected cells that were treated with isoalantolactone and/or radiation as well as the untreated controls cells were collected for the clonogenic survival assay and western blot analysis.

### Western Blot Analysis

The cells were treated with isoalantolactone and/or radiation. After 24 hours, the cells were collected and washed twice with PBS and were lysed on ice with lysis buffer as described previously [20]. Then, after the lysates were centrifuged at 12000 rpm for 20 minutes at 4°C, the supernatants were removed. Protein concentrations were determined using a NanoDrop 1000 spectrophotometer (Thermo Scientific, USA). Thereafter, 30 μg of isolated proteins was electrophoresed using SDS-PAGE (10%) and transferred onto a PVDF membrane. After blocking with 5% (w/v) non-fat milk and washing with Tris-buffered saline-Tween solution (TBST), the membranes were incubated overnight at 4°Cwith the appropriate diluted primary antibodies. The signals were detected by ECL in combination with a chemiluminescence kit and X-ray film (Millipore Corporation, Billerica, USA). Protein bands were visualized with Image J software.

### Statistical analysis

All data are shown as the mean ± standard error (SEM). All experiments were repeated at least three times. The level of significance between two or more groups was assessed by Student’s t-test or by one-way ANOVA followed by Tukey’s multiple comparisons test. *P*<0.05 was considered statistically significant.

## Results

### Isoalantolactone enhances the sensitivity of UMSCC-10A cells to radiation

**Fig 1:**
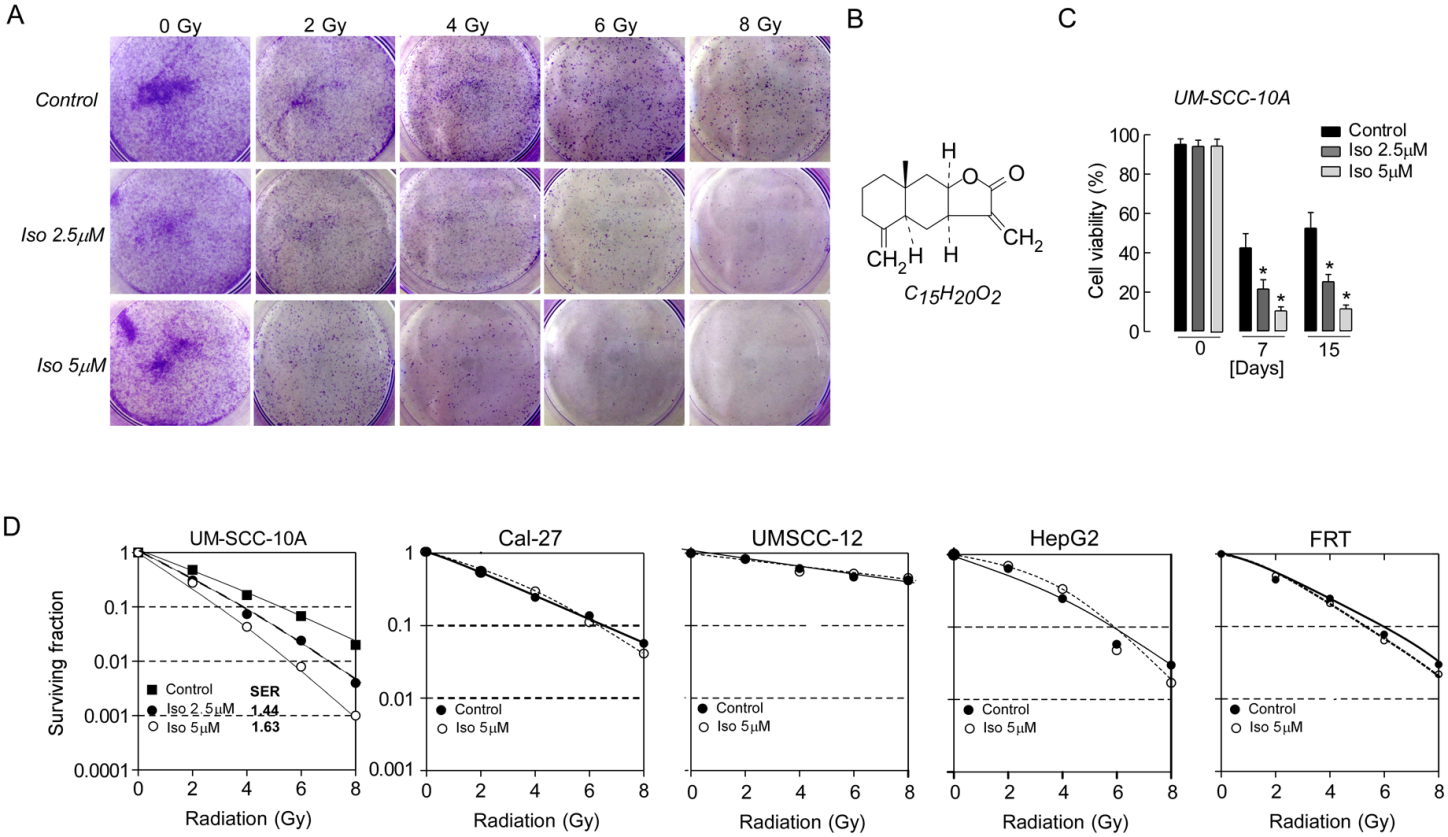
Isoalantolactone enhances the sensitivity of UMSCC-10A cells to radiation. (A) Representative images of the clonogenic survival assay. A colony formation assay was performed on UMSCC-10A cells treated with 2.5 or 5 μM isoalantolactone for 4 hours prior to radiation treatment with 0, 2, 4, 6, 8 Gy. After incubation for 14 days, the cells were stained with coomassie blue, and the colonies with more than 50 cells were counted. (B) The chemical structure of isoalantolactone. (C) Cell viability was measured using live/dead cells counted method. UMSCC-10A cells were pretreated with isoalantolactone and then following radiated 4 Gy at 0, 7, 15 days. The data are expressed as the mean±SEM from three independent experiments. *P<0.01compared with the control. (D) Radiosensitization by isoalantolactone on UMSCC-10A cells, other HNSCC cell lines (UMSCC-12 cell (radiation resistant) and Cal-27 cell), other tumor cell line (HepG2 cell) and normal cells (FRT cell). SER was calculated as the ratio of the mean inactivation dose under control conditions divided by the mean inactivation dose after isoalantolactone treated (mean ± SEM, n = 4).

In order to explore the interaction of isoalantolactone and radiation, we first needed to know whether UMSCC-10A cells were sensitive to radiation. The radiosensitivity of UMSCC-10A cells was examined by clonogenic assay after exposure to various doses of radiation (0 to 8 Gy). As shown in Fig 1A, the survival cells were reduced accompany with radiation dose increased. This suggested that UMSCC-10A cells were relatively sensitive to radiation. To determine whether a low dose of isoalantolactone can enhance the radiosensitivity of UMSCC-10A cells, a concentration much lower than the IC_50_ value was used, which generated an inhibition rate lower than 10% when given alone. Based on data from our previous study [20], doses of 2.5 and 5 μM isoalantolactone (chemical structure shown in Fig 1B) and radiation at a dose of 4 Gy were chosen to treat the cells. After radiation treated (0, 7, 15 days), cells were counted to determine the cell viability in UMSCC-10A cells. As shown Fig 1C, combination with low doses of isoalantolactone led to more cells death than treated with radiation alone (*P*<0.01). Moreover, the clonogenic assay was used to determine the extent of reproductive death caused by radiation alone or combination of radiation and isoalantolactone in UMSCC-10A cells (Fig 1A). Isoalantolactone at 2.5 and 5 μM led to a reduced number of radiation-induced colonies compared with cells treated with radiation alone. We then analyzed the SF (survival fraction) values used UMSCC-10A cell, other HNSCC cell lines (UMSCC-12 cell (radiation resistant) and Cal-27 cell), other tumor cell line (HepG2 cell) and normal cell (FRT cell), which are shown in Fig 1D. A dose-dependent radiosensitization by isoalantolactone was observed in UMSCC-10A cells with SERs (sensitivity enhancement ratio) of 1.44 and 1.63 (*P*<0.01). However, isoalantolactone could not enhance the radiosensitivity of UMSCC-12, Cal-27, HepG2 and FRT cells (*P*>0.05). These results strongly indicated that isoalantolactone could enhance the sensitivity of UMSCC-10A cells to radiation and that a relatively low dose of isoalantolactone was needed for UMSCC-10A cells.

### Isoalantolactone enhances the inhibition of radiation-induced cell proliferation of UMSCC-10A cells

**Fig 2:**
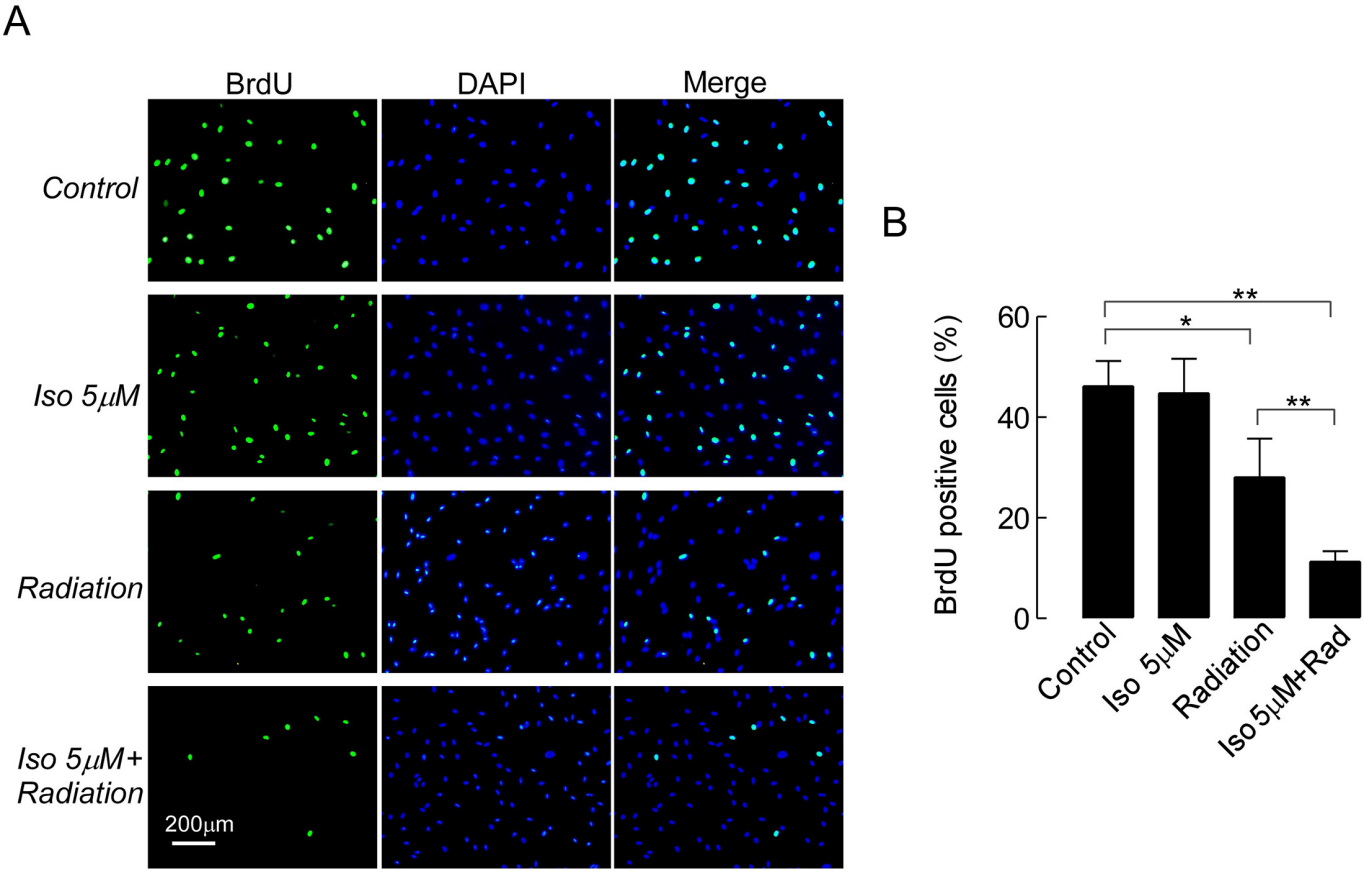
Isoalantolactone combined with radiation inhibits the proliferation of UMSCC-10A cells. (A) Representative images from a cell proliferation assay illustrate nuclear staining after the cells were treated with isoalantolactone at 5 μM for 72 hours or radiation at 4 Gy for 4 hours alone or with a combination. (B) The proliferative ability of UMSCC-10A cells was tested with BrdU incorporation assays. The results are representative of three independent experiments. Values represent the mean ± SEM, * *P*<0.05, **P<0.01compared with the control.

To evaluate whether isoalantolactone could enhance the radiation-induced inhibition of cell proliferation, we tested the proliferation of UMSCC-10A cells using a BrdU incorporation assay. As shown in Fig 2A, 5 μM isoalantolactone alone (44.8%) could not inhibit the proliferation of UMSCC-10A cells relative to the untreated cells (46.2%, *P*>0.05), whereas cells treated with radiation experienced a significant reduction in cell proliferation (28.1%, *P*<0.05). Compared with radiation or the control group, the proliferation rate of the cells treated with a combination of isoalantolactone and radiation was significantly decreased (11.3%, *P*< 0.01, Fig 2B).

### Isoalantolactone enhances radiation-induced cell cycle arrest in UMSCC-10A cells

**Fig 3:**
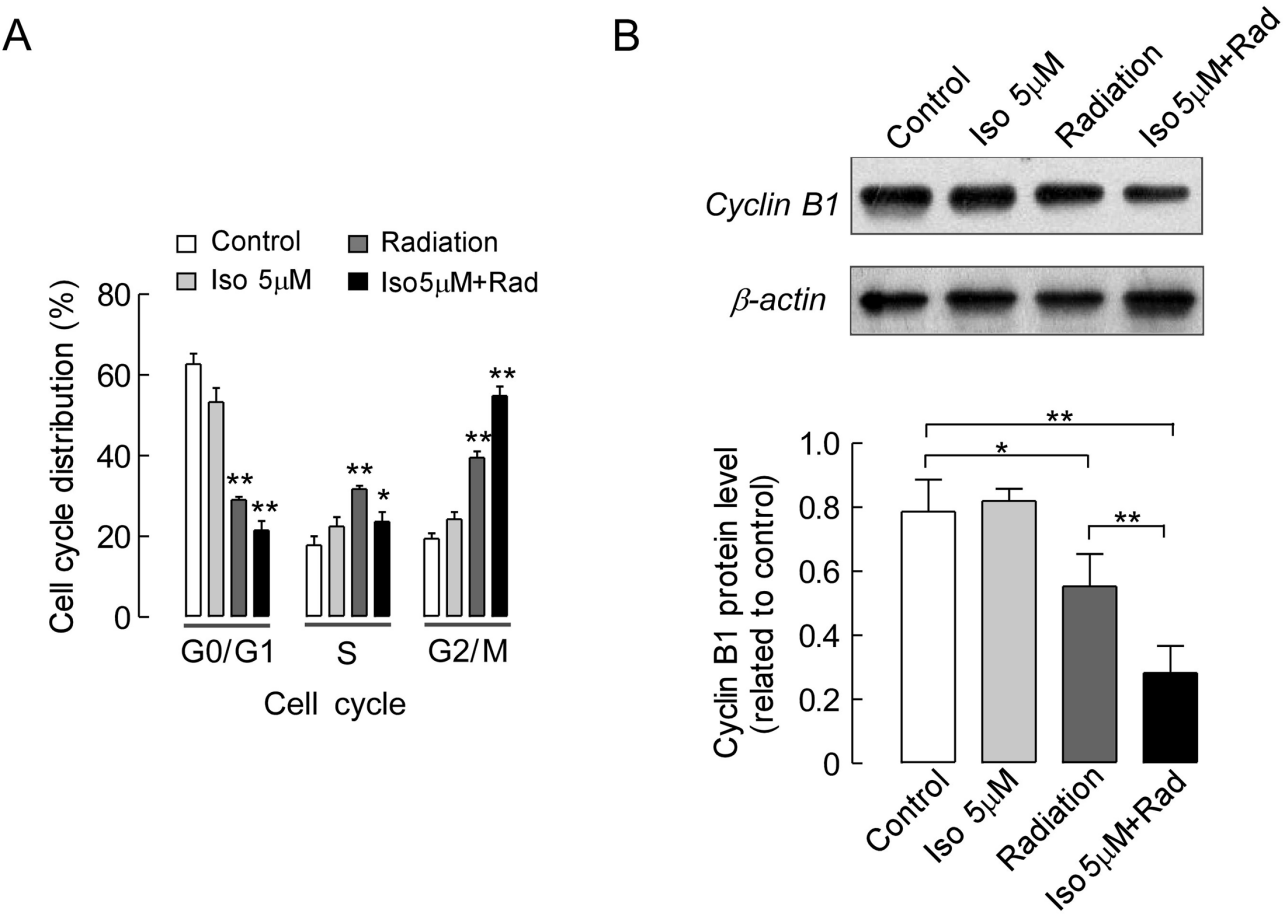
Isoalantolactone enhances radiation-induced G2/M cell cycle arrest in UMSCC-10A cells. (A) The effects of isoalantolactone and radiation alone or in combination on cell cycle distribution. The cells were treated with a combination of isoalantolactone and radiation, which isoalantolactone at a dose of 5 μM was added to the cultured cells 16 hours prior to radiation 4 Gy exposure. Those led to an increase in the proportion of cells in G2/M phase; this also led to a slight increase in the number of cells in S phase and a decrease in the number of cells in G1 phase compared with the control. (B) Representative images of Cyclin B1 protein expression by western blot analysis. β-actin was used as a control. Cells that were treated with a combination of isoalantolactone and radiation showed a significant down-regulation of Cyclin B1 expression compared with cells that were untreated and those that were treated with radiation alone. The results are presented as the mean ± SEM for three independent experiments where similar results were obtained. *P<0.05 and **P<0.01 compared with the control.

We next sought to determine if isoalantolactone in combination with radiation treatment could affect the cell cycle progression of UMSCC-10A cells. It is well known that radiation leads to breaks in the DNA of tumor cells, which arrests the cell cycle at G2/M phase and induces cell death [22]. In order to detect the DNA content during the cell cycle, we performed a flow cytometric analysis of UMSCC-10A cells treated with radiation or isoalantolactone either alone or in combination. Fig 3A shows that the DNA content of cells in S and G2/M phases of the cell cycle after treatment with isoalantolactone was not significantly different compared with that in control cells (*P*>0.05), whereas a slight decrease in DNA content was observed in cells in G0/G1 phase (*P*<0.05). The cells treated with radiation significantly accumulated in G2/M phase, and the percentage of cells in G2/M phase increased from 22.1% to 40.3% (*P*<0.01). The percentage of treated cells in S phase increased slightly from 18.0% to 30.5% (*P*<0.01), whereas the percentage of treated cells in G0/G1 phase significantly decreased from 59.9% to 29.2% (*P*<0.01) compared with control cells. However, when the cells were treated with a combination with isoalantolactone and radiation, the percentage of cells in S phase slightly decreased from 30.5% to 22.1% (*P*<0.05), while the number of cells in G0/G1 phase significantly decreased from 29.2% to 18.6% (*P*<0.05). Moreover, a marked increase in the number of cells in G2/M phase was observed (from 40.3% to 59.3%) relative to the cells treated with radiation alone (*P*<0.01). To investigate the mechanism of isoalantolactone combined with radiation, which induced cell cycle arrest in UMSCC-10A cells, we analyzed Cyclin B1 expression by western blot. As shown in Fig 3B, the expression levels of Cyclin B1 were markedly decreased in cells treated with radiation (*P*<0.05) or a combination of radiation and isoalantolactone (*P*<0.01) compared with control cells. In cells treated with a combination of isoalantolactone and radiation, Cyclin B1 expression was greatly reduced relative to its expression in cells treated with radiation alone (*P*<0.01). Those results indicated that isoalantolactone and radiation can block cell cycle progression at G2/M phase.

### Isoalantolactone enhances the apoptotic effect induced by radiation in UMSCC-10A cells

**Fig 4:**
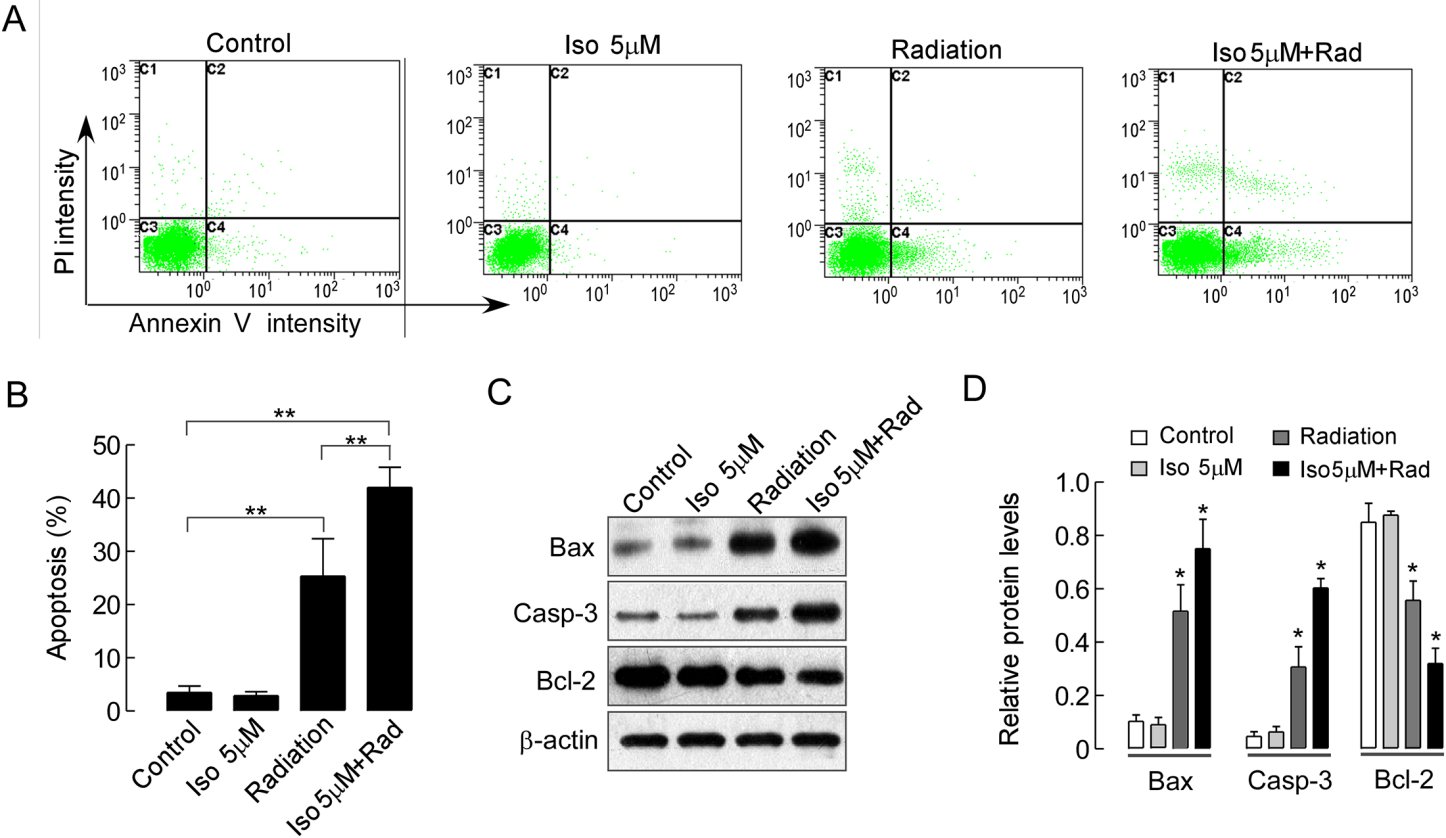
Isoalantolactone increases radiation-induced apoptosis in UMSCC-10A cells. (A) Cells were treated with isoalantolactone or radiation individually or with a combination of isoalantolactone and radiation. Isoalantolactone at a dose of 5μM was added to the cultured cells 16 hours prior to radiation 4 Gy exposure. The effect on apoptosis was analyzed by flow cytometry using Annexin V-FITC and propidium iodide (PI) staining in UMSCC-10A cells. (B) Isoalantolactone enhanced radiation-induced apoptosis in the UMSCC-10A cells. The data are expressed as the means ± SEM of three independent experiments where similar results were obtained. **P<0.01 compared with the control or radiation alone. (C) Bax, Bcl-2, Cleaved-caspase 3 and Pro-caspase 3 protein expression in UMSCC-10A cells was determined by Western blot. (D) The quantification of Bax, Bcl-2, Cleaved-caspase 3 and Pro-caspase 3 protein expression is shown. β-actin was used as a control. Values represent the mean ± SEM, * *P*<0.01, compared with the control or radiation alone.

While apoptosis induces cell death, it also enhances radiosensitivity [23]. We next assessed whether increased radiosensitivity by isoalantolactone was associated with enhanced induction of cell apoptosis. Annexin V-conjugated FITC and PI staining were performed to evaluate the percentage of apoptotic cells in each group. As shown in Fig 4A, the level of apoptosis was increased in cells treated with radiation (*P*<0.01) compared with the control group. However, treatment with 5 μM isoalantolactone alone could not induce apoptosis of UMSCC-10A cells (*P*>0.05). The number of apoptotic cells was significantly increased by the combination of radiation and isoalantolactone compared with radiation alone or no treatment (*P*<0.01, Fig 4B), which suggested that isoalantolactone may enhance radiation-induced apoptosis. To further confirm the above result, some indicators of cellular apoptosis were measured. The results in Fig 4C demonstrate that the expression levels of Bax and Cleaved-caspase 3 proteins were greatly increased by the combined treatment of radiation and isoalantolactone compared with control cells (*P*<0.01) or treatment with radiation alone (*P*<0.01), whereas the level of Bcl-2 and Pro-caspase 3 protein was significantly decreased (Fig 4D). However, Bax, Bcl-2, Cleaved-caspase 3 and Pro-caspase 3 expression in cells treated with isoalantolactone alone was not significantly different compared with that in control cells (*P*>0.05, Fig 4D). Taken together, these data suggested that isoalantolactone contributes to the ability of radiation to enhance the induction of apoptosis.

### Effect of isoalantolactone radiosensitization on the inhibition Erk1/2 phosphorylation in UMSCC-10A cells

**Fig 5:**
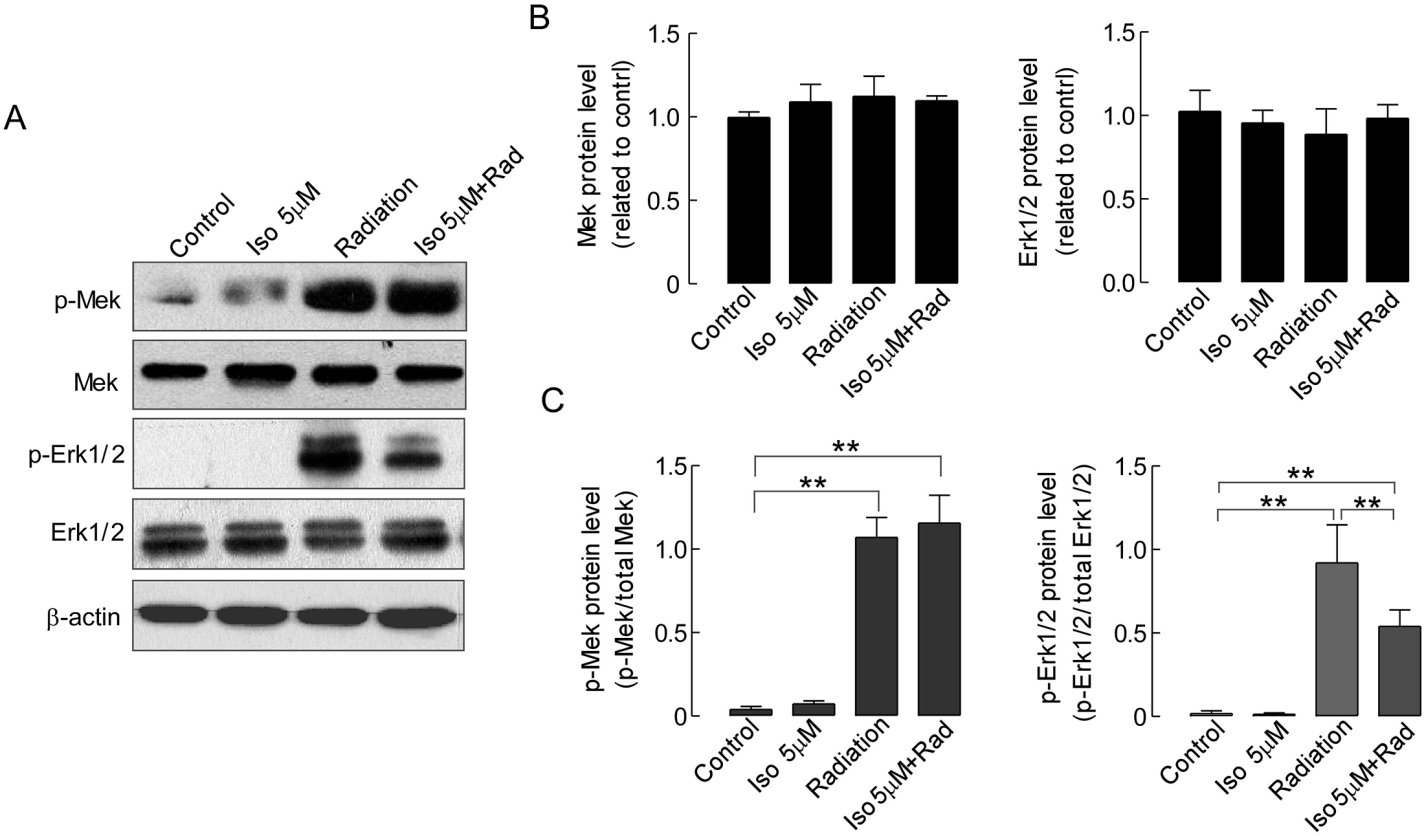
Isoalantolactone inhibits radiation-induced Mek and Erk1/2 protein phosphorylation in UMSCC-10A cells. (A) Whole-cell protein extracts were prepared from UMSCC-10A cells that were treated with radiation and isoalantolactone alone or in combination. Isoalantolactone at a dose of 5 μM was added to the cultured cells 16 hours prior to radiation 4 Gy exposure. The blot was probed with antibodies against Mek, Erk1/2, p-Mek, p-Erk1/2 and β-actin. (B) Mek and Erk1/2 protein expression relative to that of β-actin were assessed by densitometric analysis. p-Mek and p-Erk1/2 were expressed as the radio of p-Mek/total Mek and p-Erk1/2/total Erk1/2. ** *P*<0.01, compared with the control or radiation alone.

The Mek/Erk1/2 signaling pathway plays a key role in the regulation of tumor cell proliferation, cell cycle progression and survival [24]. Moreover, radiation at low doses can induce the activation of the Mek/Erk1/2 survival pathway in tumor cells, which results in instant cellular proliferation to compensate for cell loss [16]. This has been thought to be one of the major reasons why tumors are resistant to radiation. With this aim, we investigated the possibility that isoalantolactone might enhance the sensitivity of UMSCC-10A cells to radiation via blockage of Mek/Erk1/2 signaling pathway activation. We first examined the total Erk1/2 protein expression level and its phosphorylation levels after the cells were treated with radiation alone or in combination with isoalantolactone for 24 hours (Fig 5A). No marked changes in the total protein expression of Erk1/2 were found following radiation or combination treatment with radiation and isoalantolactone (*P*>0.05, Fig 5B). Compared with control cells, the phosphorylation of Erk1/2 was significantly increased in cells treated with radiation alone (*P*<0.01). However, the combination treatment of radiation and isoalantolactone greatly reduced the phosphorylation of Erk1/2 protein compared with radiation alone (*P*<0.01, Fig 5C). Moreover, we also tested the cells treated with isoalantolactone alone and observed fewer changes in the phosphorylation level of Erk1/2 (*P*>0.05). Those data suggested that isoalantolactone inhibits radiation-induced Erk1/2 phosphorylation in UMSCC-10A cells.

To further confirm the specific inhibition of Erk1/2 phosphorylation by isoalantolactone, we then examined the total protein level as well as the phosphorylation level of Mek, which functions upstream of Erk1/2 in the Ras-Raf-MEK-ERK signaling pathway [25]. As shown in Fig 5A, few changes were observed in the total protein level of Mek in UMSCC-10A cells (Fig 5B). Although the phosphorylation of Mek level were increased in cells treated with radiation and combination of isoalantolactone and radiation compared with control cells (*P*<0.01, Fig 5C), no significant difference in the phosphorylation level of Mek were observed between both groups (*P*>0.05). These data further supported that isoalantolactone can specifically inhibit the phosphorylation of Erk1/2 in UMSCC-10A cells.

**Fig 6:**
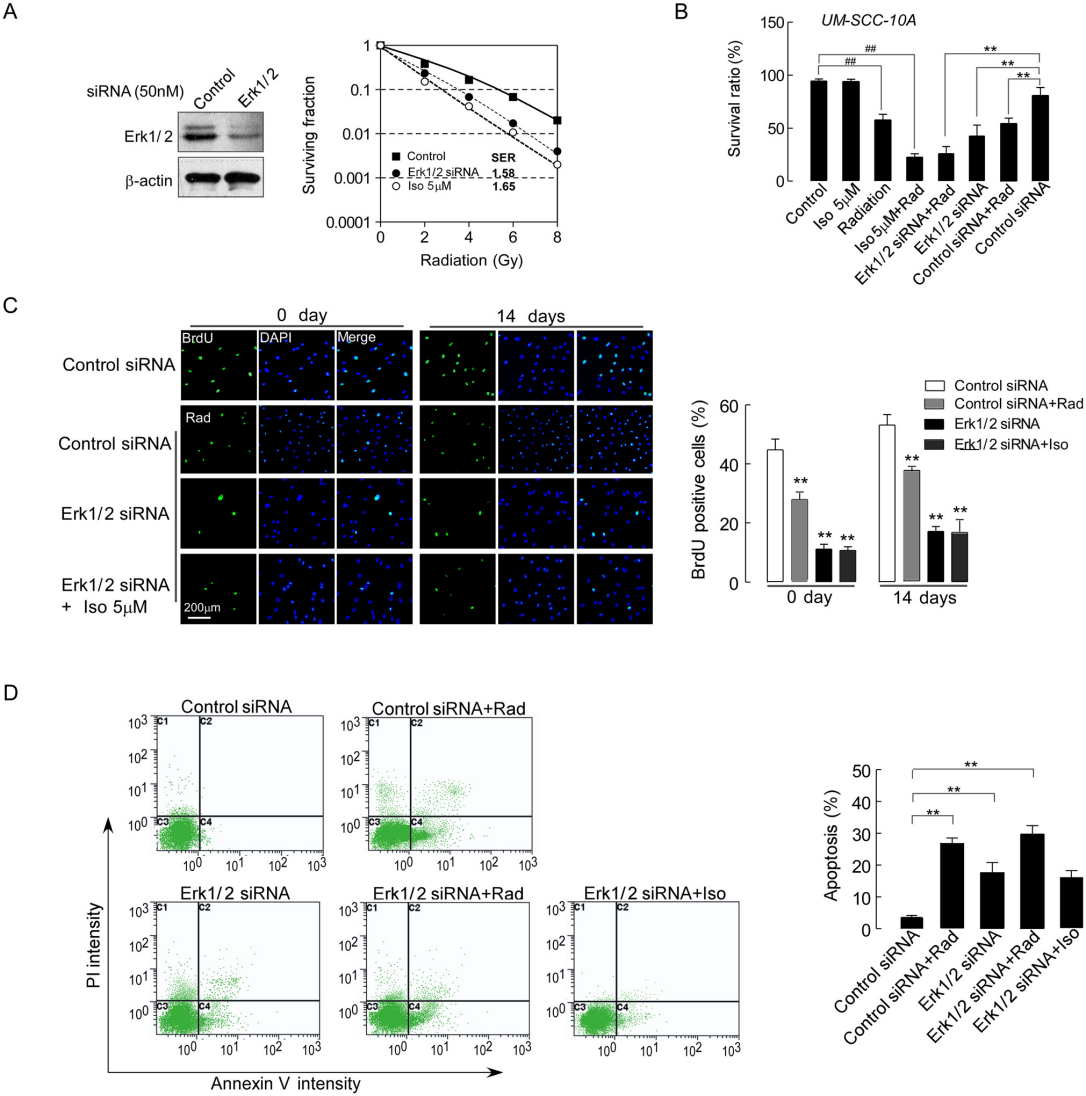
Radiosensitization by siRNA-mediated knockdown of Erk1/2 in UMSCC-10A cells. (A) Radiosensitization by Erk1/2 knockdown. Cells were transfected using Lipofectamine 2000 and siRNA that targets Erk1/2. Seventy-two hours later, one portion of the cells was reserved for immunoblotting (top) and the other portion was plated for the clonogenic assay (bottom, mean ± SEM, n = 3). (B) The cell survival ratio was measured in UMSCC-10A cells that were treated with isoalantolactone, radiation alone or with a combination of the isoalantolactone and radiation after siRNA-mediated knockdown of Erk1/2. Isoalantolactone at a dose of 5 μM was added to the cultured cells 72 hours prior to radiation 4 Gy exposure. The results are presented as the mean±SEM from three independent experiments.^##^ *P*<0.01, compared with the control. * *P*<0.05, compared with the Erk1/2 siRNA. ** *P*<0.01, compared with the control siRNA. (C) Representative images for a cell proliferation assay at difference time after cells by Erk1/2 silencing treated with isoalantolactone at 5 μM or combination of isoalantolactone and radiation 4 Gy. The results are presented as the mean±SEM from three independent experiments. ** *P*<0.01, compared with the control cells. (D) Apoptosis was analyzed by flow cytometry using Annexin V-FITC and propidium iodide (PI) staining in UMSCC-10A cells. Cells were transfected using Erk1/2 siRNA and treated with radiation or isoalantolactone individually. The data are expressed as the means ± SEM of three independent experiments where similar results were obtained. **P<0.01 compared with the control siRNA.

In addition, we also analyzed the effect of siRNA on Erk1/2 in UMSCC-10A cells by western blot. As shown in Fig 6A left, after the knockdown of Erk1/2 to approximately 40–50% of the original level, we found that the UMSCC-10A cells with Erk1/2 knockdown demonstrated increased radiosensitivity with an SER of 1.58 (Fig 6A, right). Thus, the Erk1/2 levels correlated with radioresistance. Similarly, isoalantolactone radiosensitization was also significantly increased with an SER of 1.65 (P<0.05, Fig 6A, right). We also tested the survival ratio of cells after Erk1/2 knockdown and radiation treatment. A similar survival ratio was found between the Erk1/2 knockdown cells with radiation and cells treated with a combination of isoalantolactone and radiation (P>0.05, Fig 6B). Furthermore, we performed cell proliferation assay on UMSCC-10A cells with Erk1/2 knockdown and combined with isoalantolactone following radiated. As shown in Fig 6C, cells proliferation were inhibited by Erk1/2 knockdown relative to control cells (P<0.01). Although cells with Erk1/2 knockdown would slightly proliferation after radiation 14 days, isoalantolactone cannot further inhibit the proliferation of UMSCC-10A cells (*P*>0.05). Cells apoptosis also were analyzed on cells with Erk1/2 knockdown induced by radiation or isoalantolactone as Fig 6D shown. Cells with Erk1/2 siRNA induce slightly apoptosis and enhance the level of apoptosis induced by radiation compared with cells treated with radiation only (P<0.05). However, isoalantolactone 5 μM cannot enhance apoptosis induced by cells with Erk1/2 siRNA. This indicated that isoalantolactone combined with radiation can specifically inhibit the cell proliferation by activation of the Erk1/2 signaling pathway. Thus, isoalantolactone may be a potent inhibitor of Erk1/2-mediated radiation resistance of UMSCC-10A cells.

## Discussion

Radiotherapy is one of the mainstream approaches for the treatment of HNSCC, as it exerts its effects on local tumor control and reduces disease recurrence [6]. The purpose of radiotherapy is to kill tumor cells as efficiently as possible without the induction of side effects in normal cells. However, radiotherapy alone for the treatment of HNSCC is always associated with resistance to radiation. Therefore, it is urgent to find an agent with the ability to specifically sensitize tumor cells to radiotherapy, as this would be an important step toward the improvement of outcomes in the treatment of patients with advanced HNSCC.

Plant-derived compounds possess the characteristics of safety, effectiveness and low toxicity, which is why plants are one source of antitumor therapies in some cancer types [18, 19, 26, 27]. Moreover, many studies have demonstrated that natural compounds from plants could enhance antitumor effects of radiation in tumor cell lines [21, 28–30]. Isoalantolactone (chemical structure shown in Fig 1B) is known as a naturally occurring compound that exerts a potent effect on tumor cell proliferation [17]. In our previous publication [20], we demonstrated that isoalantolactone selectively exerted potent anti-cell proliferative effects in UMSCC-10A cells. The growth-inhibitory effect of isoalantolactone was thought to induce intrinsic mitochondrial apoptosis. In addition, normal mouse splenocytes that were treated with isoalantolactone did not display a significantly cytotoxic effect in vitro. Moreover, consistent with our research, Khan et al. [19] reported that isoalantolactone did not cause any detectable acute or chronic toxicity in the liver and kidney in vivo. However, to date, research has not yet focused on the efficacy of radiosensitization of tumor cells by isoalantolactone.

In the present study, we intended to investigate the radiosensitizing effect of isoalantolactone as a radiosensitizer of UMSCC-10A cells and to further reveal the molecular mechanisms of its action. We first demonstrated the radiosensitivity of UMSCC-10A cells in a dose-dependent manner by a classic clonogenic assay (Fig 1A). The dose of radiation required for 50% inhibition was approximately 4 Gy. Thus we choose this dose of radiation for our experiments. The ideal cancer radiosensitizer would lead to the use of a reduced radiation dose and fewer side effects. Our results showed that isoalantolactone at lower doses 2.5 or 5 μM can effectively enhance the sensitivity of UMSCC-10A cells to radiation (SER = 1.44 or 1.63) according to the gold standard clonogenic assay (Fig 1D) and can synergistically enhance the anti-cancer proliferative effects of radiation (Fig 2) in UMSCC-10A cells. Moreover, we also analyzed the radiosensitivity effects of isoalantolactone on other HNSCC cell lines including radiation resistant cells, other tumor cell line and normal cell. As expected, isoalantolactone cannot enhance the sensitivity of those cells (Fig 1D). This is best evidence that isoalantolactone might possess the potency of a radiosensitizer for UMSCC-10A cells.

It is well known that radiation can cause cell cycle arrest at G2/M phase through the activation of p53 (the tumor suppressor protein), which inhibits tumor cell growth [22]. As previously mentioned, isoalantolactone at a high dose of 25 or 50 μM can induce cell cycle arrest, but not at G2/M phase. Rather, it can only enhance the accumulation of UMSCC-10A cells in G1 phase of the cell cycle, which leads to cell death according to a cell cycle assay. In the present study, we observed the synergistic effect of low-dose isoalantolactone and radiation on the cell cycle in UMSCC-10A cells. Moreover, the more abnormal cells were blocked in G2/M phase of the cell cycle (Fig 3A). The accumulation of cells in G2/M phase may be related to the down-regulation of endogenous Cyclin B1. To gain further insight into the molecular mechanisms that underlie the efficacy of the combination of isoalantolactone and radiation-induced G2/M arrest in UMSCC-10A cells, we tested Cyclin B1 expression. Western blot analysis showed that the combination of isoalantolactone and radiation significantly suppressed the expression of the cell cycle-related protein Cyclin B1 in UMSCC-10A cells (Fig 3B).

In addition, the occurrence of radioresistance during radiotherapy is associated with the up-regulation of Bcl-2 expression in primary HNSCC tissues [31]. Moreover, Yang et al. [32] further confirmed by siRNA assay that Bcl-2 knockdown could enhance the sensitivity of radioresistant HNSCC cells to radiation. According to our previous data, isoalantolactone at IC_50_ doses could induce the down-regulation of Bcl-2 expression and the up-regulation of Bax expression; this would activate the intrinsic mitochondrial apoptosis pathway, which would in turn result in cell death of UMSCC-10A cells. The present studies demonstrate that the treatment of cells with a combination of low-dose isoalantolactone and radiation might further reduce the expression of Bcl-2 and increase the expression of Bax, which would lead to an increased Bax/Bcl-2 ratio; moreover, Cleaved-caspase 3 expression would also increase and result in enhanced radiation-induced apoptosis. Caspase-3 has been identified as an important mediator of apoptosis in mammalian cells. This notion is supported by our study in that treatment with isoalantolactone along with radiation significantly enhanced radiation-induced apoptosis in these cells according to a FACS assay, as shown in Fig 4. This might be one of the ways in which isoalantolactone functions as a radiosensitizer.

The Mek/Erk1/2 pathway is an important regulator of cell proliferation. Activation of the Mek/Erk1/2 survival signaling pathway is involved in radioresistance in various tumor cells [16, 33], which leads to instant cellular proliferation in order to compensate for cell loss caused by the stress of radiation. With this aim mind, our mechanistic study revealed that UMSCC-10A cells were sensitized to radiation by siRNA silencing of Erk1/2 (Fig 6). Moreover, we found that isoalantolactone could enhance the radiosensitization of UMSCC-10A cells via blockage of Erk1/2 phosphorylation (Fig 5C). Erk1/2 is a target of Mek and the activity of Erk1/2 required activation of the upstream kinase Mek. We also observed that the radiation-induced increase in the phosphorylation of Mek protein, which is a key upstream molecule in the Ras-Raf-Mek-Erk signaling pathway, primarily results in cell proliferation and survival [25]. However, cells treated with a combination of isoalantolactone and radiation did not result in the effective inhibition of Mek phosphorylation (Fig 5C). Of note, we further found that isoalantolactone cannot prevent the proliferation of UMSCC-10A cells with Erk1/2 knockdown following radiation. It was clear in UMSCC-10A cells that the inhibition of Erk1/2 phosphorylation might be the major mechanism of isoalantolactone radiosensitization.

## Conclusion

In summary, we demonstrate here that isoalantolactone enhanced radiation-induced apoptosis, cell cycle arrested and reduced the cell proliferation of UMSCC-10A cells via specifically inhibited the phosphorylation of Erk1/2. Thus a low concentration of isoalantolactone may be used to overcome the resistance of UMSCC-10A cells to radiation and may be a promising radiosensitizer in cancer therapy.
